# Decentralized clinical trials: A comprehensive analysis of trends, technologies, and global challenges

**DOI:** 10.1371/journal.pdig.0001191

**Published:** 2026-01-16

**Authors:** Sara Kijewski, Claire McBride, Eric Owens, Elsa Bernheim, Effy Vayena

**Affiliations:** Health Ethics and Policy Lab, Department of Health Sciences and Technology, ETH Zurich, Zürich, Switzerland; Instituto Politécnico Nacional Escuela Superior de Medicina: Instituto Politecnico Nacional Escuela Superior de Medicina, MEXICO

## Abstract

Decentralized clinical trials (DCTs), particularly in the U.S., gained substantial attention during the COVID-19 pandemic, enabling trial activities to be conducted from participants’ homes or local healthcare facilities despite restrictions and lockdowns. Regardless of the growth in interest, many facets of the DCT landscape remain unexplored or nascent in their development. This study aims to explore the key characteristics and development of the U.S.-registered DCT landscape, adoption patterns across various clinical contexts, and the role of digital technologies. We analyzed 1370 decentralized trials from ClinicalTrials.gov, collected using a broad DCT-keyword search. The data were screened and coded manually, and analyzed descriptively for temporal trends, purpose of decentralization, intervention type, geographic representation, and digitalization. Our findings align with previous reports of a growing, heterogeneous landscape of DCTs, with behavioral interventions appearing more suitable for decentralization than other types of interventions. Notably, most DCTs still focus on evaluating decentralized methods rather than merely implementing them in their investigations. Often, studies integrate digital tools either as the interventions themselves or to enable the digital delivery of study activities. Although the trial registry used is U.S.-based, and a U.S. partner is part of more than 50% of the studies identified, many trials are done in multiple countries or countries outside of the U.S. (42%). Among these trials, the data revealed considerable differences, with digitalized DCTs in this sample concentrated in high-income countries. Despite rapid growth in DCTs, our findings suggest the presence of a field in development, very much focused on establishing a methodological foundation. To unlock the potential of DCTs locally and globally, four critical areas demand further attention: digital equity, regulatory frameworks for diverse technologies, establishment of methodological validation processes, and further research on barriers to implementation.

## Author summary

Since the COVID-19 pandemic, decentralized clinical trials (DCTs) have been widely discussed, particularly in the U.S. DCTs enable clinical trial activities to be done at participants’ homes or other local locations, rather than requiring all activities be in-clinic. The DCTs landscape is still developing and underexplored. This study examines characteristics and development patterns of DCTs. We used a keyword search to collect trial data on 1370 DCTs from ClinicalTrials.gov. We manually reviewed the data and assigned trials to categories. We analyzed the data to understand changes over time and the dynamics of decentralization and digitalization. We also analyzed the types of trials and regions represented. Like previous studies, we found a growing and varied landscape of DCTs. Most DCTs still aim to evaluate decentralized methods rather than simply implement them. DCTs use digital tools as interventions themselves or for digital delivery of trial activities. We observed differences in the digitalization of DCTs across regions, with most digitalization in high-income countries. Our study suggests that the DCTs field is still in development, aiming to establish a methodological foundation before widespread use. Future work should address digital equity, regulatory frameworks, validation of DCT methods, and roadblocks to implementation.

## Introduction

Randomized controlled trials (RCTs) have been, and continue to be, considered the “gold standard” of clinical trial design [1]. However, as many trials require physical presence at hospitals or clinical treatment sites, the availability and willingness of participants to travel to these sites pose recurrent issues for investigators (and participants). The COVID-19 pandemic underscored this inherent constraint in RCTs, as restrictions on physical contact and mobility were needed to contain the virus. This appeared to catalyze interest in decentralized clinical trials (DCTs), which are trials in which “some or all of a clinical trial’s activities occur at locations other than traditional clinical trial sites” [2]. This may include participants’ homes, local healthcare facilities, or nearby laboratories [3]. Whether as fully remote or hybrid trials, DCTs aim to integrate research participation into participants’ daily lives [4].

The promise of decentralization extends well beyond necessity during the pandemic. By eliminating geographic barriers to trial participation, DCTs can expand representation and promote participant diversity in trial populations [5–7]. Broader access to, and higher inclusivity of, clinical research may benefit the study of issues like rare diseases and/or conditions affecting populations with limited mobility [2]. Further, DCTs can potentially reduce costs through streamlined data collection and analysis [7] or shorten drug development timelines [8]. These potential benefits have generated strong enthusiasm for DCTs, prompting some scholars to suggest that DCTs are “the new normal in clinical trials” [6,9]. Additionally, digital technologies are often considered instrumental to DCTs [9,10] and are commonly integrated throughout the research process [11,12]. The Trials@Home research consortium, for example, explicitly defines DCTs as trials “that make use of digital innovations and other related methods to make them more accessible to participants” [13]. Digital technologies can be integrated across all research phases, from social media recruitment and online screening to telemedicine interventions and remote data collection and monitoring with wearable devices, with emerging AI-based tools potentially improving these capabilities further.

Empirical studies document substantial growth in DCTs over time [14,15], with scholars describing the field as “burgeoning” [9]. Decentralization of trials also appears to be well-received among many participants, with research on trial participants’ experiences indicating high acceptance of decentralized methods [16]. A survey of public and participant perceptions of decentralization demonstrated that nearly 80% of the respondents wish to have the option of remote study procedures [14]. Despite this enthusiasm for decentralization, the research on decentralized trial design and implementation still remains in its early stages [15]. There are also numerous and diverse factors challenging the decentralization of trials and shaping how decentralization manifests in practice. For instance, a systematic review of the literature identified 34 distinct technological, regulatory, societal, and logistical obstacles to DCT implementation, ranging from immature digital infrastructure and regulatory uncertainty to privacy concerns and digital access inequalities [16]. Implementation challenges include practical aspects such as decentralized drug delivery and management [11,17], verifying participant identity [17], and the clinical validation of digital tools [11,18]. While large-scale geographically dispersed sampling of trial participants can expand access to a diverse group of trial participants, it may also raise concerns about investigator oversight, data quality, and participant safety [4]. The transition from traditional clinical trials to DCTs requires substantial adaptation across multiple research operations such as skills, training, and composition of research groups, infrastructure, study administration, data collection and management, quality control, ethics, and safety [19]. A survey of clinical trials research staff and participants found that more than 70% are concerned about an increased burden on sites with decentralization, participant trust and safety, and insufficient site support for the use of technology for decentralized trials [20]. Another survey revealed that decentralized trial activities are adopted less frequently than conventional activities, with many research sites lacking the time, training, and financial resources to implement decentralized activities effectively [21].

The gap between the theoretical promise and the practical reality of DCTs raises fundamental questions about the actual state of the field. This study systematically analyzed the current landscape to answer the following critical questions: What are the key characteristics of decentralized trials, and how has the landscape of U.S.-registered DCTs evolved? How are DCTs implemented across clinical or geographical contexts? What is the role of digital technologies in DCTs?

This study examines these questions through a systematic empirical analysis of 1370 decentralized trials from 2000 to 2023, aiming to provide critical insights for researchers, sponsors, regulators, and policymakers. Expanding our understanding of this landscape can help inform standards and regulations to ensure safe and effective DCTs and aid in realizing the benefits of this approach for U.S. (and likely global) clinical research.

## Data and methodology

### Data collection

We collected the data for the analysis from ClinicalTrials.gov, the largest online database of clinical trials research studies in the world [22], which is managed by the U.S. National Library of Medicine. The data collection was performed in two steps. First, to locate relevant studies, we identified a set of keywords associated with DCTs and decentralized trial elements and searched for trials using these keywords. Second, we manually screened the trials according to our inclusion criteria described below (see also S1 Fig in the supplementary material for a visual of the data collection and screening process).

The keyword search was performed based on 19 common keywords linked to DCTs (see below). These were identified based on the Trials@Home glossary [13], as well as three key academic papers [5,9,23]. We use the term DCT to refer to trials with decentralized elements, which includes both fully decentralized and hybrid trials, aiming to conduct a comprehensive analysis of the scope and modes of decentralization in clinical trials. We decided to implement a broader keyword search based on two observations. First, although DCTs have been defined in the literature, the terminology used to describe such trials is marked by pluralism, as also noted by others [9]. Currently, there is no standardized, shared terminology for decentralized methods [24,25]. Not only are terms such as “decentralized”, “remote”, and “virtual” trials used interchangeably, but the terms “decentralized elements” or “decentralized trial activities” have also emerged to specifically describe trial activities that “are organized around the trial participants and conducted away from investigative sites” [22]. Our preliminary search also revealed inconsistent use of terminology in records in the database, with trials not necessarily using descriptors such as “DCTs” or “hybrid trials” despite using decentralized elements. We identified and defined the final list of keywords through an iterative process which resulted in the use of these keywords: “decentralized” AND “trial”, “decentralized”, “decentralized trial”, “decentralized clinical trial”, “remote trial” “digital trial”, “home trial”, “hybrid trial”, “virtual trial”, “remote monitoring”, “remote consent”, “eConsent”, “remote recruitment”, “remote intervention”, “social media” AND “recruitment”, “televisit”, “teleconference” AND “trial”, “telemedicine” AND “trial”, and “web-based trial”.

The final database search was conducted on 15.04.2024, producing a dataset of 2130 clinical trials (2367 before the removal of duplicates in Excel based on the NCT-number). We further manually screened this data and excluded all withdrawn and suspended trials (n = 45), trials that lacked an actual start date (n = 219), and those that started after 31.12.2023 (n = 103). This cutoff point was chosen to provide a temporal analysis that only includes complete calendar years. Finally, we excluded all trials lacking decentralized elements (n = 383), resulting in a final sample of 1370 trials.

For each of the trials, we directly exported the following information from the database: trial title, summary, full description, conditions, interventions, primary and secondary outcomes, sponsors, collaborators, sex, age, enrollment, study type, start date, and location. The extracted dataset was largely complete, and, where possible, we manually filled in any missing data with relevant study information available elsewhere in the database. To retrieve the information on the actual start date and actual enrollment, which was not included in our initial dataset, we created a web scraper that collected only this data. The program was implemented with Python 3.13.2 and 3.12.3 (Python Software Foundation, Wilmington, DE, USA), with the final extraction taking place on 25.11.2024. The full dataset is found in the supplementary file S1 Data.

### Data preprocessing

#### Clinical conditions.

To better understand the clinical contexts in which DCTs are integrated, we categorized all trials by the clinical conditions they address. We based this categorization on the conditions mentioned in the dataset, sorting them into higher-level categories. The list of conditions and the higher-level categories in which they are included can be found in the supplementary material in S1 Table. There are 18 global condition categories and one for the trials in which none of the condition keywords were found, labelled “Other”, which includes 77 trials. These trials were commonly described with highly general condition terms like “multimorbidity” or “healthy”, or very specific, less common condition terms such as “Mucopolysaccharidosis III-A” or “Arachnophobia”.

#### Decentralization.

An initial review of the trials included in our dataset revealed that decentralized design elements were incorporated in studies with differing motivations. To reflect this in our analysis, we coded each study according to whether it was **implementing a decentralized design** or **evaluating decentralization** (see S2 Fig and S2 Table in the supplementary material for the full coding tree and codebook). Specifically, studies that implemented decentralized design elements as a matter of course were coded as implementing decentralized design(s), while those that aimed to evaluate the efficacy, feasibility, or acceptability of decentralized trial activities, often in comparison to standard, centralized trial protocols, were coded as evaluating decentralization. In cases where both aspects were present, the classification as evaluation took precedence, as evaluation necessarily entails implementation (e.g., testing a disease-specific online platform), whereas implementation does not necessarily entail evaluation (e.g., utilizing Zoom for behavioral therapy sessions).

#### Digitalization.

To examine how digital tools are integrated in DCTs, we also coded the trials depending on whether and to what extent they integrate digital technologies, distinguishing between the integration of digital health tools and digital delivery of trial activities with general digital tools. First, all studies were coded as either involving a **digital health tool** or not, depending upon whether digital health tools were described in the study title, study overview, or study plan. We defined use of a “digital health tool” as studies involving devices, platforms, apps, or other digital services *for healthcare purposes*. Our understanding of the term aligns with its use in the literature, including Soobiah et al.’s definition of digital health interventions as “health services delivered electronically through formal or informal care” [26]. Second, we coded all trials without any digital health tools based on whether they used **non-health digital tools** for the delivery of trial activities (apart from a digital health tool) or only used **analog** means. For example, if study personnel communicated with participants via email or social media, or if questionnaires were distributed online, we coded the study as involving non-health digital tools in the delivery of trial activities. Though rare in our dataset, fully analog studies included those in which, for example, study materials were sent, and data were returned entirely by mail. Importantly, the modes of digitalization were not mutually inclusive or exclusive and were conceptualized as representative of different points on a spectrum of how specialized and crucial they were for the trials. Digital health tools tended to predominantly play a central role in DCTs, representing a deeper integration of digitalization, as the focus of trials involving a digital health tool in most cases revolved specifically around those digital elements. Digital delivery of trial activities was understood to play a more enabling, optional, role in DCTs – simplifying trial delivery and supporting decentralization of trial elements, although the trial could conceivably be run in a centralized, analog way. This would, for example, include a behavioral trial that is delivered over videoconferencing. Notably, most studies that involved a digital health tool also entailed digital delivery of trial activities.

### Data analysis

To answer the research questions, we analyzed the data using descriptive statistics, presenting observed proportions without inferential statistics or uncertainty measures. The descriptive statistics and figures were produced using Stata SE/17.0 (StataCorp, College Station, TX, United States) and Python 3.12.3 (Python Software Foundation, Wilmington, DE, United States).

## Results

### Overview

**Fig 1:**
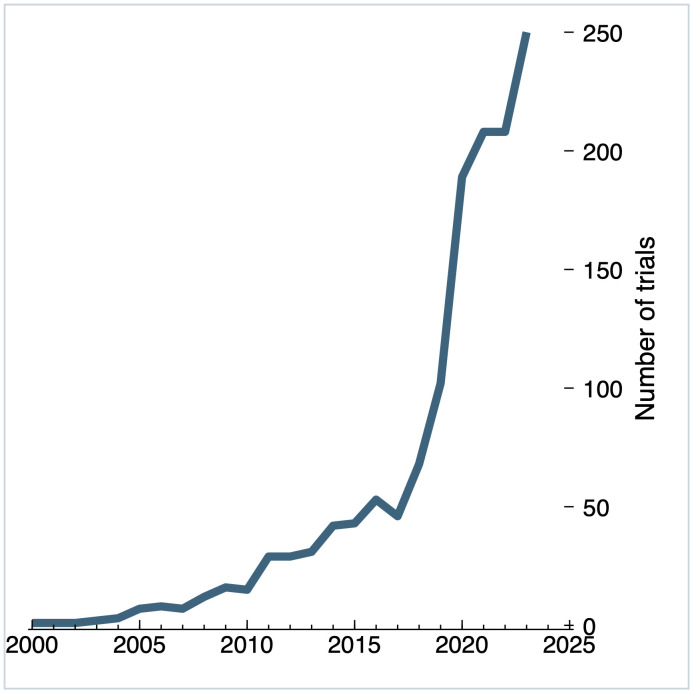
Number of registered decentralized trials from 2000 - 2023, n = 1370.

The final dataset includes trials that started between 2000 and 31.12.2023. Fig 1 illustrates a steady growth in DCTs over this period. There was a noticeable increase in the number of trials from 2017, growing from 46 trials in 2017 to 102 in 2019, a twofold increase over two years. From 2019, the number of DCTs grew rapidly, rising from 102 in 2019 to 189 in 2020. This coincides with the COVID-19 pandemic, which was officially declared on 11 March 2020 by the World Health Organization. By the end of 2023, the number of registered trials grew to 250.

880 studies included in our dataset provided data on the actual number of enrolled participants. The studies exhibited considerable variation regarding enrolled participants, ranging from one participant in some studies (e.g., NCT05086120, NCT04644315) to 64,666 participants (e.g., NCT03259373). On average, the trials had a mean of 538 and a median of 85 participants. A vast majority included both male and female participants (1247 trials). Of the remaining trials, 95 focused on women and 27 on men. Most trials included adults and elderly people (999 trials), while 111 focused on all age categories, 75 on children, and 43 on elderly people, specifically.

**Fig 2:**
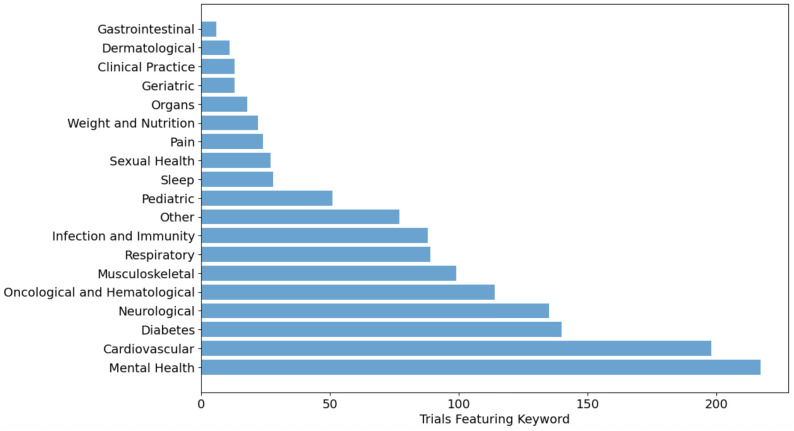
Clinical conditions targeted by the clinical trials, n = 1370.

Fig 2 displays the clinical conditions addressed by the trials across the aforementioned 19 condition categories (including the “Other” category, n = 77). Mental health-related or cardiovascular conditions were, by far, the main conditions addressed, with 217 and 198 trials devoted to each of these types of conditions, respectively. Trials for diabetes and neurological conditions were also well represented in the dataset, with almost 140 trials devoted to each. Oncological and hematological and musculoskeletal conditions followed, with around 100 related trials each.

**Fig 3:**
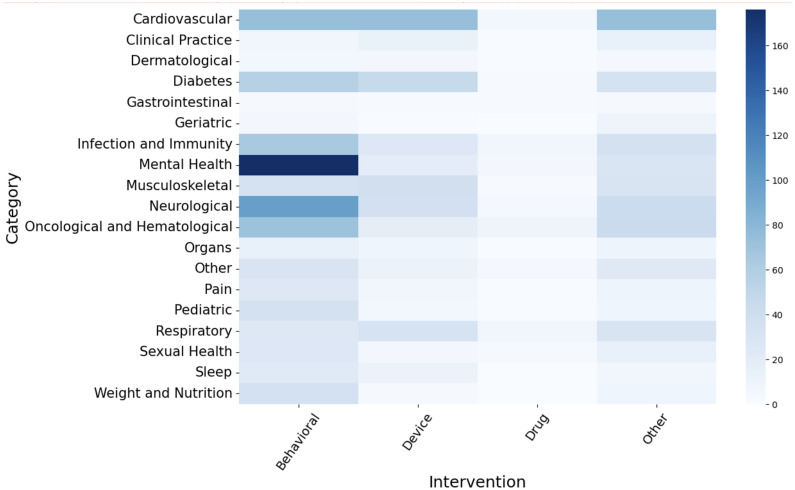
Clinical conditions by type of intervention, n = 1323.

Of the 1370 studies, interventional trials made up 89% (n = 1218) and observational trials made up 11% (n = 152). The majority of interventional studies were classified as behavioral interventions (47%), “Other” (26%), or “Device” (21%). Trials examining a wide range of interventions, from exercise interventions to health education programs, fell into the “Other” category. Drug trials and trials for procedures represented only 3% and 2% of the DCTs, respectively. Fig 3 illustrates the most frequent intervention types by clinical conditions. The most common types of intervention tested in DCTs were behavioral interventions for mental health issues, behavioral interventions for neurological issues, behavioral interventions, devices, and “other” interventions for cardiovascular diseases, and behavioral interventions for infections and immunity, oncological and hematological issues, and diabetes.

**Fig 4:**
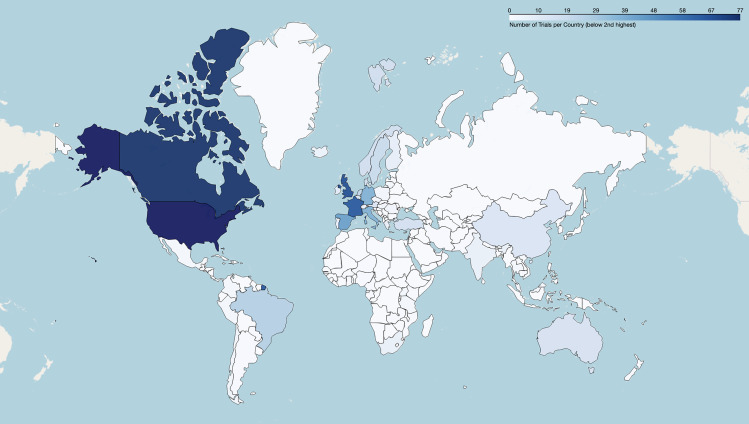
Geographic distribution of trials with decentralized elements, includes multi-country trials; n = 1370. Made with Natural Earth. Free vector and raster map data @ naturalearthdata.com.

Fig 4 displays the geographic distribution of the sampled trials. The DCTs examined in this study took place in 75 different countries (see S3 Table in the supplementary material for a full list of trial location countries). One trial lacked location information. The data reveal that a large share of trials is concentrated in a few countries, mainly the U.S. (799), followed by Canada (77), the UK (69), and France (66). While the U.S. and European countries are overrepresented, a smaller number of studies were run in several African (23), South American (35), and Asian countries (65). It should be noted that the sum is not equal to 1370, as some trials involved more than one country. Of trials run in a single country (n = 1325), a disproportionate number were run in one of 40 high income countries (1228), while a smaller number of trials were run in 13 upper-middle income countries (76) and 17 lower-middle and 5 low income countries (20) respectively (for this country classification, we rely on the World Bank classification [27]). 45 of the trials are multi-country trials, with four including lower-middle- and low-income countries (e.g., NCT04038632, NCT01805752, NCT01430936, NCT04810650).

### Purpose of decentralization

**Fig 5:**
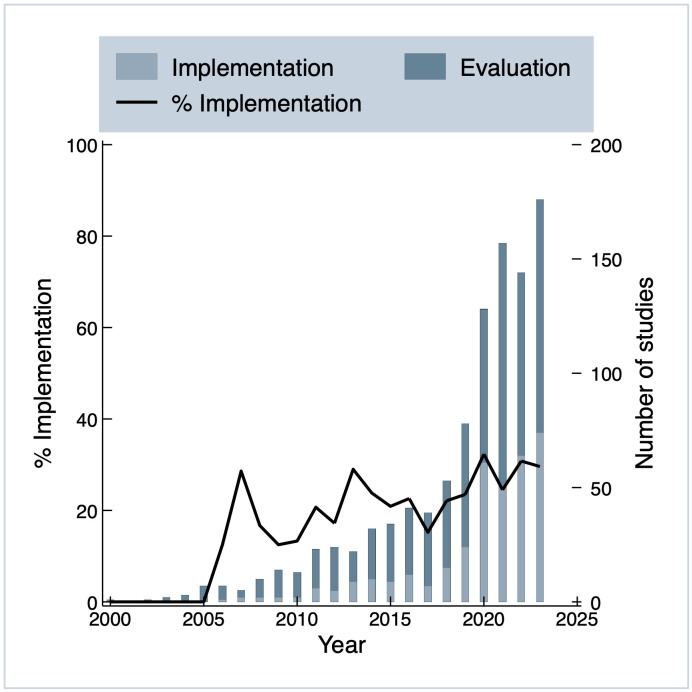
Trials by purpose of decentralization over time, n = 1370.

Analyzing how decentralization manifests in the clinical trials, our data indicate that 26% of the examined trials merely implemented decentralized elements to investigate the impact of interventions, whereas 74% evaluated the feasibility, efficacy, or acceptability of decentralized activities themselves. Fig 5 displays the growth in trials by type of implementation over time. It shows that since 2000, the share of evaluation trials has been consistently larger than the share of trials implementing decentralized activities. Both groups of trials have grown over time, and especially after 2017. The number of implementation trials jumped between 2019 and 2020 (154% growth), with only moderate growth after 2020. Evaluation trials have also continued to increase in numbers, reaching an all-time high number of studies in 2023 (176 trials).

The share of implementation trials has fluctuated within the total number of studies (see line in Fig 5). Despite this variation, the proportion of implementation studies appears to be slightly increasing, possibly indicating a slight maturation of the field.

Among trials that evaluate decentralization, we identify three main study design approaches: 1) comparing decentralized to centralized elements, 2) comparing decentralized elements with other decentralized elements, and 3) assessing the feasibility or efficacy of decentralized elements. For instance, some trials (e.g., NCT05033548) compared remote monitoring applications for post-organ transplantation to standard aftercare and in-clinic follow-up. Other trials (e.g., NCT01848080) compared telerehabilitation to home visits for increasing access to post-stroke rehabilitation services, while others again (e.g., NCT04039698) evaluated the feasibility of telemedicine monitoring and video consultations for monitoring for hepatitis C treatment. An analysis of these evaluation studies (n = 1014) shows that the most common study design was to compare centralized and decentralized elements (43% of the studies), whereas 22% compared different decentralized activities, 29% examined the efficacy or feasibility of decentralized elements/activities, and 6% mixed designs.

### Digitalization

Our analysis of the role of digital technologies in decentralized studies reveals that only 5% of the studies in our sample did not include digital technologies in any way (see Table 1). Of those that integrate digital technologies, the data shows that more than half of the studies integrate digital health technologies (53%), whereas 42% use digital tools to deliver trials. A typical example of a trial that delivers trial activities digitally is a behavioral intervention delivered with telehealth (e.g., NCT02070874). An example of an intervention with a digital health tool is the use of a smartphone weight management app to promote weight loss (e.g., NCT04453072). When split by type of trial, we find that interventional trials are slightly more likely to integrate digital health tools (50%) compared to digital delivery of trial activities (45%), whereas observational trials integrate a high share of digital health tools (74%) compared to digital delivery of trial activities (23%).

**Table 1:** Share of DCTs by role of digital technologies and type of trial.

	All (n = 1370)	Interventional (n = 1218)	Observational (n = 152)
No digital technologies	5.0%	5.3%	2.6%
Digital health tools	52.7%	50.0%	74.3%
Non-health digital tools	42.3%	44.**7**%	23.0%

The specificity and intended uses of digital health tools varied across trials. For instance, some trials used apps specifically designed to measure or monitor trial variables (e.g., in NCT02463682, DiAs, the study’s smartphone-based medical platform, helped monitor glucose levels and adjust an artificial pancreas system). Others used more widely available tools, like tablets for participants to track measurements and send messages (see NCT04142710, in which participants receive tablets for answering health questions and submitting measurements), or email to communicate about tasks or questions (as in NCT03247608, in which daily emails inform participants about daily trial tasks). The use of digital technologies appears to include both health-specific and generic commercial tools and apps. Some trials leveraged non-health-specific platforms like Facebook to message participants or host chat groups (e.g., in NCT02037490, participants were part of a Facebook group that facilitated social interaction and sharing of an interventional video activity), while others provided clinical-grade, non-commercial apps for tasks like speech recording (as in NCT05276349). While we typically distinguish between generic and health-specific digital tools, some trials implemented commercial tools with adapted, specialized functionality (i.e., healthcare-specific models or versions), in which case they were coded as digital health tools. For instance, some trials utilized Zoom for Healthcare as a means of telemedicine communication (see NCT04639557).

Despite the potential of AI-tools, and in particular those using machine learning (ML) in clinical trials, our analysis showed that, in actuality, relatively few AI/ML tools are being used in DCTs. Specifically, of the 1370 included trials, only 23 (less than 2%) explicitly mentioned the use of AI tools. All of these were in studies involving digital health tools. 15 of these examined the use of AI elements specifically (e.g., in NCT04948632, a study-specific telehealth platform with integrated AI was compared with the same platform without the integrated AI). The remaining 8 cases utilized AI-based tools in the implementation of the studies (e.g., in NCT06237998, ML techniques were utilized to analyze the collected data for predictive factors).

**Fig 6:**
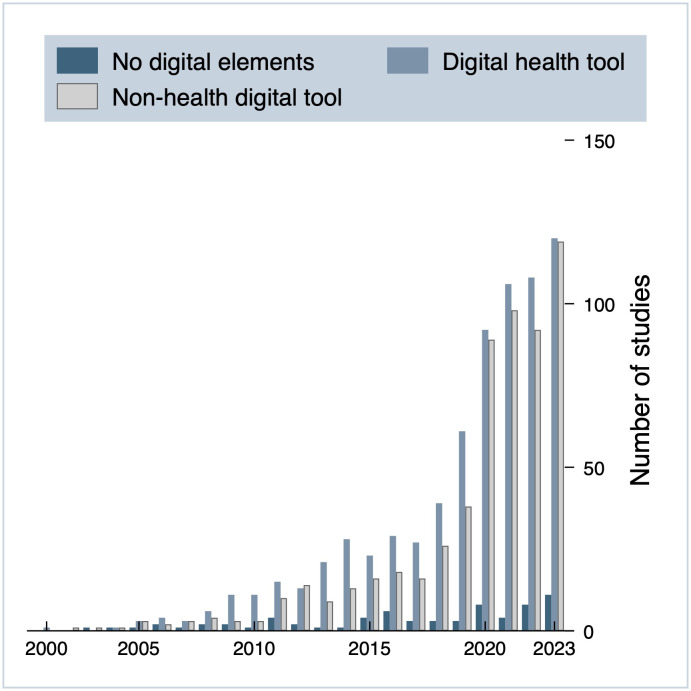
Trials by digitalization from 2000-2023, n = 1370.

Fig 6 displays the development in trials by digitalization between 2000 and 2023. While non-digital decentralized trials have seen a modest increase over time, trials incorporating digital technologies have grown rapidly. Except for in 2012, use of digital health tools has represented the most frequent form of digitalization in DCTs over time. From 2019 to 2020, we observed a sharp rise in the number of trials utilizing digital technologies to support trial delivery, almost reaching the same number of trials integrating digital health tools. Since then, trials utilizing digital tools for trial delivery have become more frequent, with a particularly high number of trials in 2023.

**Fig 7:**
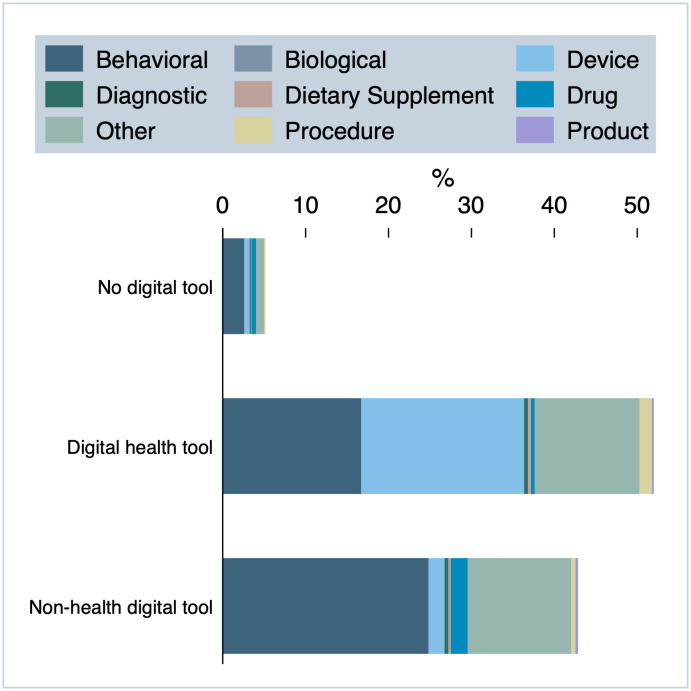
Use of digital technologies by type of intervention, n = 1323.

Fig 7 displays the variation in digital technology use across different types of interventions in decentralized trials. Notably, behavioral trials represent a dominant share of the trials utilizing digital technologies for trial delivery. Among trials that incorporate digital health tools, device-focused trials are highly represented, reflecting the integration of a digital investigational product. Both types of digital technologies appear to be common in the intervention category “Other”. Studies that do not utilize any digital technologies include behavioral trials, those testing conventional devices, drug trials, and “Other” trials.

Further analysis of the level of DCT digitalization over time, split by the income-ranked status of the trial location for single country trials (see S3 Table and S3 and S4 Figs in the supplementary files), indicates that the sharp increase in digitalized DCTs is concentrated heavily in high income countries, while upper-middle income countries experience a less steep, but marked increase over time, particularly over the past decade.

### The interplay between digitalization and decentralization

Examining both the purpose of decentralization and the level of digitalization, we can identify six categories of trials in the data (see S4 Table). While there is a considerable overlap between the categories, as suggested in the previous section, there is a small share of studies that entail decentralized elements without integrating digital tools. 2% of the studies do not integrate any digital tools but implement decentralized elements, whereas 3% of the studies evaluate decentralized elements without using any digital tools. These include, for example, a study of the impact of mineral-enriched salts on the neurological health of stroke survivors (NCT02910427) or a study comparing music-with-movement intervention to usual care on patients with dementia or mild cognitive impairment (NCT03575026). Trials implementing decentralized elements tend to utilize digital tools to support trial delivery (14% of all trials), with a smaller share integrating digital health tools (10%). Evaluation trials more frequently (43%) integrate digital health tools, but also often utilize non-health digital tools to support trial delivery (28%).

### Discussion and conclusion

Our study uncovered a rapidly expanding landscape of decentralized trials, consistent with previous findings of strong growth in DCTs in recent years, accelerating in 2020 [28,29]. While some of this growth likely reflects patterns of evolving reporting incentives and requirements [30,31], our data confirms an exceptional surge in the number of studies coinciding with the COVID-19 pandemic between 2019 and 2020, which has continued post-pandemic. Considering developments in clinical trials generally, which have grown steadily since 2006, with numbers growing by more than 20,000 studies annually since 2014 and 30,000 studies since 2018 [32], DCTs appear to be part of a broader increase in all trial activity. However, the stark growth in DCTs from 2017 remains striking. One possible explanation is the introduction of the 21st Century Cures Act, which may have helped lay part of the foundation for decentralized trials, requiring the FDA to develop frameworks for evaluating the use of real-world data and evidence, including data collected using digital health technologies [33].

The adoption of decentralized trials is distributed heterogeneously across clinical contexts, strongly suggesting that not all clinical trials are equally suitable for decentralization. Mental health, cardiovascular, neurological diseases, and diabetes appear to be particularly amenable to decentralized approaches, often integrating behavioral interventions and devices. Behavioral interventions are clearly the most conducive to decentralization, representing nearly half of the DCTs in our sample, followed by device trials (20%). This pattern suggests that trials for which core activities can be conducted remotely (such as behavioral therapy, device monitoring, data collection), are most suitable for decentralization, whether it is through digital or analog means. The low number of drug and procedural trials may reflect specific risks, inherent challenges, and practical barriers preventing their adoption. This raises questions about whether DCTs can truly transform drug development or if they will remain confined to specific types of interventions. Our findings of continued growth in DCTs contrast with a recent study narrowly focusing drug trials, documenting declining numbers of DCTs post-2021 [34], attributing this to sponsors avoiding risks of experimental methods and an unrealized potential for cost reduction and efficiency.

Although the ongoing growth in DCTs may seem to suggest a maturing of the field, a closer examination of the data produces a more nuanced picture. The higher prevalence of evaluation studies compared to implementation studies may reflect continued development, exploration and establishment of methodologies for decentralization. This is a natural and expected stage in the adoption of new research approaches. To ensure safety, efficacy, and feasibility of decentralized trials across diverse contexts, decentralized research methods need to be systematically evaluated before they are widely implemented. Once approaches are validated, DCTs hold promise to potentially increase inclusivity and accessibility of clinical trials [7] and provide benefits to participants and study teams alike [4,9]. While success metrics may vary based on trial aims and context, ethical and practical performance measures for DCTs may include decreased burden on participants [4], real-world data collection [4], cost reductions [8], and inclusion of underrepresented demographics [7]. Stakeholders considering implementing DCTs should consider these metrics and determine how they may align with study goals and ethics considerations.

Furthermore, our findings illustrate that the decentralization and digitalization of clinical trials do not overlap entirely. Whereas most DCTs integrate digital technologies, their implementation varies substantially. A small minority of studies do not adopt any digital technologies, while others integrate digital tools in two ways: either enabling remote delivery of trial activities or as integral elements of the trial itself. The results indicate that the technologies facilitating and supporting DCTs comprise a highly diverse ecosystem integrating both healthcare-specific (e.g., telehealth platforms) and general-purpose (e.g., Facebook, Skype, and commercial messaging apps) digital technologies and devices. Trials implementing decentralized elements tended to favor non-health digital tools for the delivery of trial activities, possibly a sign that tools for digital delivery have matured beyond needing feasibility testing or face less scrutiny or fewer regulatory hurdles for operational implementation. The minimal adoption of AI/ML tools identified in our study echoes other research on the integration of AI in clinical trials [35] and stands in stark contrast to the general optimism around AI for healthcare. This gap between AI optimism and the pattern we observe may reflect a lack of regulatory frameworks or standardized procedures for the validation of such tools, and practical obstacles to adoption. While their functional capacities and usability increase, further research is needed to explore how such tools may impact areas like patient privacy, data storage, sustainability, and patient-physician relationships.

Beyond these implementation challenges, global disparities in DCTs add another critical dimension to our findings, with concentration of digitalized decentralized trials mainly in high-income countries. This pattern may underscore differences in research infrastructure across the globe, particularly regarding technology use and infrastructure, financial resources, and expertise. Further, this points to a risk of an increasing global divide rather than realizing the promise of DCTs to increase access, reduce health inequities, and democratize trial implementation and participation. Interestingly, some trials run in lower-resourced, rural settings were decentralized using no digital technologies or less advanced technologies, including decentralization of care through local clinics or primary health settings (e.g., NCT04038632) or remote monitoring via phonelines (e.g., NCT04108143). This demonstrates the feasibility of context-sensitive DCT design as a potential path to enable the democratized benefits of DCTs while work to address global digital inequity continues. Importantly, these disparities may also be related to other factors. For example, DCTs addressing mental health conditions made up the majority of studies in our dataset, and mental health stigma is a significant challenge facing low- and middle-income countries [36]. Future research should investigate these dynamics further to develop evidence that can inform practical recommendations to address these disparities. Despite only analyzing a U.S.-based registry of DCTs, we believe that such trends may be present to a greater degree than what we were able to explore. Further research should focus on expanding the scope of trials to incorporate additional trial registries and potentially compare the implementation of DCTs with non-DCTs across jurisdictions to shed more light on these dynamics.

Together, these findings illustrate a rapidly developing field characterized by substantial growth and technological diversification while simultaneously confronted with fundamental questions about implementation, validation, and equity. We identify four key areas that demand further attention from researchers, regulators, and policymakers. First, to realize the promise that decentralization can expand access to clinical research and prevent further reinforcing global disparities in clinical research, investments in digital research infrastructure, context-specific technology selection, and capacity-building for local research teams are needed. Nebie and colleagues’ [37] work in Sub-Saharan Africa underscores the need for adapting trial technologies to local contexts and investing in continuous development of local expertise to enable and ensure continuity of decentralized trials in such contexts.

Second, regulatory frameworks will need adaptation to address the technological diversity in digitalized DCTs. Among other issues, the use and blend of healthcare-specific and general-purpose tools raise questions about appropriate governance frameworks and standards to ensure the protection of patient information. While specialized platforms like Zoom for Healthcare comply with the U.S. Health Insurance Portability and Accountability Act (1996) to ensure the safe transmission and storage of personal health information in the U.S., other consumer applications (e.g., FaceTime, WhatsApp, etc.) may not have the appropriate safeguards in place [38]. This is especially pertinent for recent advances in AI such as large language models. While the safe transmission of information is crucial for the broader medical field, and some professional associations (e.g., FMH Swiss Medical Association [39]) have begun to issue guidance on the use of general-purpose tools for patient communication in medical practice, the extent of possible remote communication, data collection and monitoring in DCTs presents higher risks and may require specialized governance frameworks.

Third, methodological validation continues to be essential if DCTs are to transition from experimental approaches to standard research practice and requires systematic evaluation of the appropriateness of decentralization approaches across contexts. As also argued by others [37,40], digital health technologies must be assessed for their suitability for clinical conditions, local context, study design, and end-user needs before integration in clinical studies. Sehrawat and colleagues [40] propose practical solutions, such as qualification programs for technologies and allowing technology adoption across multiple studies to streamline validation. Finally, while the above considerations are crucial to drive the field forward, a better understanding of the challenges researchers and research sites face when conducting DCTs is crucial to developing strategies to address the gap between evaluation and implementation studies.

This study has several limitations. First, the reliance on ClinicalTrials.gov for trial records may skew the results due to the overrepresentation of U.S.-based studies in the database. The findings on global distribution of such trials should therefore be interpreted with caution, as the patterns may reflect registration patterns rather than actual differences in adoption among countries. Other trial registries may reveal variations in the patterns of decentralized trial adoption. Second, the lack of standardization in reporting and, at times, incomplete trial records both complicated and/or made it impossible to code certain features of the studies. For example, studies self-identifying as “decentralized” (e.g., NCT06087276, NCT02832063) often did not specify which elements were decentralized. Our coding was necessarily based on the available information, which varied considerably in detail across records. Where information was absent, we tried to retrieve it from elsewhere on the website. Naturally, some cases remained ambiguous after further scrutiny; cases that contained discrepancies or ambiguous elements were discussed among all parties to come to a coding decision.

As DCTs evolve, it would be valuable for ClinicalTrials.gov to incorporate standardized fields to document decentralization. Such standardization would not only facilitate more robust research on decentralized trials but also provide clearer guidance for researchers designing new decentralized studies. To effectively leverage the promise of digitally enabled DCTs, further efforts are required to define contextually appropriate, ethical, and technically validated best practices for decentralization. As the field continues to grow, its greatest scientific and practical contributions to clinical research may still be ahead if decentralized methods can manage the transition from development to standard research practice.

## Supporting information

S1 Data Full dataset. (ZIP)

S1 Fig Flow diagram illustrating data collection and screening. (TIF)

S1 Table Full list of clinical conditions. (DOCX)

S2 Fig Coding decision tree. (TIF)

S2 Table Codebook. (DOCX)

S3 Table List of trial location countries with country status. (DOCX)

S3 Fig Digitalization over time by income rank of study location. (TIF)

S4 Fig Number of trials at three levels of digitalization by country status over time. (TIF)

S4 Table Exemplars illustrating variation of DCTs across decentralization and digitalization dimensions. (DOCX)

## References

[1] Hariton E, Locascio JJ. Randomised controlled trials - the gold standard for effectiveness research: Study design: randomised controlled trials. BJOG. 2018;125(13):1716. doi: 10.1111/1471-0528.15199 29916205 PMC6235704

[2] U.S. Department of Health and Human Services Food and Drug Administration. Conducting Clinical Trials With Decentralized Elements Guidance for Industry, Investigators, and Other Interested Parties [Internet]. 2024. Available from: https://www.fda.gov/media/167696/download

[3] Center for Drug Evaluation, Research. U.S. Food and Drug Administration. FDA; 2023 [cited 2023 Dec 6]. The Evolving Role of Decentralized Clinical Trials and Digital Health Technologies. Available from: https://www.fda.gov/drugs/news-events-human-drugs/evolving-role-decentralized-clinical-trials-and-digital-health-technologies

[4] de Jong AJ, van Rijssel TI, Zuidgeest MGP, van Thiel GJMW, Askin S, Fons-Martínez J. Opportunities and challenges for decentralized clinical trials: European regulators’ perspective. Clin Pharmacol Ther. 2022;112(2):344–52. 35488483 10.1002/cpt.2628 PMC9540149

[5] Hanley DF Jr, Bernard GR, Wilkins CH, Selker HP, Dwyer JP, Dean JM. Decentralized clinical trials in the trial innovation network: value, strategies, and lessons learned. J Clin Transl Sci. 2023;7(1):e170. 10.1017/cts.2023.597 PMC10465321 37654775

[6] Dorsey ER, Kluger B, Lipset CH. The new normal in clinical trials: decentralized studies. Ann Neurol. 2020;88(5):863–6. 32869367 10.1002/ana.25892

[7] Jean-Louis G, Seixas AA. The value of decentralized clinical trials: Inclusion, accessibility, and innovation. Science. 2024;385(6711):eadq4994. doi: 10.1126/science.adq4994 39172847

[8] DiMasi JA, Smith Z, Oakley-Girvan I, Mackinnon A, Costello M, Tenaerts P, et al. Assessing the Financial Value of Decentralized Clinical Trials. Ther Innov Regul Sci. 2023;57(2):209–19. doi: 10.1007/s43441-022-00454-5 36104654 PMC9473466

[9] Vayena E, Blasimme A, Sugarman J. Decentralised clinical trials: ethical opportunities and challenges. Lancet Digit Health. 2023;5(6):e390–4. doi: 10.1016/S2589-7500(23)00052-3 37105800 PMC10129131

[10] Harmon DM, Noseworthy PA, Yao X. The Digitization and Decentralization of Clinical Trials. Mayo Clin Proc. 2023;98(10):1568–78. doi: 10.1016/j.mayocp.2022.10.001 36669937 PMC12056663

[11] Van Norman GA. Decentralized Clinical Trials: The Future of Medical Product Development? JACC Basic Transl Sci. 2021;6(4):384–7. doi: 10.1016/j.jacbts.2021.01.011 33997523 PMC8093545

[12] Copland RR, Hanke S, Rogers A, Mpaltadoros L, Lazarou I, Zeltsi A, et al. The Digital Platform and Its Emerging Role in Decentralized Clinical Trials. J Med Internet Res. 2024;26:e47882. doi: 10.2196/47882 39226549 PMC11408899

[13] Trials@Home Glossary [Internet]. Trials@Home; 2019 [cited 2024 Apr 3]. Decentralized Clinical Trials. Available from: https://trialsathome.com/glossary/decentralised-clinical-trials/

[14] Richards DP, Queenan J, Aasen-Johnston L, Douglas H, Hawrysh T, Lapenna M, et al. Patient and public perceptions in Canada about decentralized and hybrid clinical trials: “it’s about time we bring trials to people.” Ther Innov Regul Sci. 2024;58(5):965–77. 38904884 10.1007/s43441-024-00665-y PMC11335844

[15] McCarthy MW, Lindsell CJ, Rajasingham R, Stewart TG, Boulware DR, Naggie S. Progress toward realizing the promise of decentralized clinical trials. J Clin Transl Sci. 2024;8(1):e19. doi: 10.1017/cts.2023.706 38384913 PMC10879994

[16] Rogers A, De Paoli G, Subbarayan S, Copland R, Harwood K, Coyle J. A systematic review of methods used to conduct decentralised clinical trials. Br J Clin Pharmacol. 2022;88(6):2843–62. 34961991 10.1111/bcp.15205 PMC9306873

[17] Hernandez AF, Lindsell CJ. Ensuring virtual vigilance in decentralized clinical trials. JAMA. 2025;333(2):119–20. 39565601 10.1001/jama.2024.22640

[18] Khozin S, Coravos A. Decentralized Trials in the Age of Real-World Evidence and Inclusivity in Clinical Investigations. Clin Pharmacol Ther. 2019;106(1):25–7. doi: 10.1002/cpt.1441 31013350

[19] Tenaerts P, Hernandez AF, Lipset C. Clinical trial site readiness for decentralized trials - fitting trials into today’s world. J Clin Transl Sci. 2024;8(1):e43. 10.1017/cts.2024.17 PMC10928696 38476244

[20] Barge R, Floody P. Comparison of Participant and Site Perceptions of Decentralized Clinical Trials in the USA. Mayo Clin Proc Digit Health. 2025;3(2):100201. doi: 10.1016/j.mcpdig.2025.100201 40568604 PMC12190979

[21] The Association of Clinical Research Professionals. Delivering on the Promise of Decentralized Trials: Unexpected Perspectives from Clinical Research Professionals. 2022 Nov. Available from: https://acrpnet.org/delivering-on-the-promise-of-decentralized-trials-unexpected-perspectives-from-clinical-research-professionals

[22] de Jong AJ, Grupstra RJ, Santa-Ana-Tellez Y, Zuidgeest MGP, de Boer A, Gardarsdottir H, et al. Which decentralised trial activities are reported in clinical trial protocols of drug trials initiated in 2019-2020? A cross-sectional study in ClinicalTrials.gov. BMJ Open. 2022;12(8):e063236. doi: 10.1136/bmjopen-2022-063236 36038171 PMC9438113

[23] Ghadessi M, Di J, Wang C, Toyoizumi K, Shao N, Mei C, et al. Decentralized clinical trials and rare diseases: a Drug Information Association Innovative Design Scientific Working Group (DIA-IDSWG) perspective. Orphanet J Rare Dis. 2023;18(1):79. doi: 10.1186/s13023-023-02693-7 37041605 PMC10088572

[24] Dulko D, Kwong M, Palm ME, Trinquart L, Selker HP. From a decentralized clinical trial to a decentralized and clinical-trial-in-a-box platform: Towards patient-centric and equitable trials. J Clin Transl Sci. 2023;7(1):e236. doi: 10.1017/cts.2023.629 38028335 PMC10663768

[25] Santa-Ana-Tellez Y, Lagerwaard B, de Jong AJ, Gardarsdottir H, Grobbee DE, Hawkins K, et al. Decentralised, patient-centric, site-less, virtual, and digital clinical trials? From confusion to consensus. Drug Discov Today. 2023;28(4):103520. doi: 10.1016/j.drudis.2023.103520 36754144

[26] Soobiah C, Cooper M, Kishimoto V, Bhatia RS, Scott T, Maloney S, et al. Identifying optimal frameworks to implement or evaluate digital health interventions: a scoping review protocol. BMJ Open. 2020;10(8):e037643. doi: 10.1136/bmjopen-2020-037643 32792444 PMC7430416

[27] How does the World Bank classify countries? – World Bank Data Help Desk [Internet]. [cited 2025 May 8]. Available from: https://datahelpdesk.worldbank.org/knowledgebase/articles/378834-how-does-the-world-bank-classify-countries

[28] Park J, Huh KY, Chung WK, Yu K-S. The landscape of decentralized clinical trials (DCTs): focusing on the FDA and EMA guidance. Transl Clin Pharmacol. 2024;32(1):41–51. doi: 10.12793/tcp.2024.32.e2 38586122 PMC10990725

[29] Sato T, Mizumoto S, Ota M, Shikano M. Implementation status and consideration for the globalisation of decentralised clinical trials: a cross-sectional analysis of clinical trial databases. BMJ Open. 2023;13(10):e074334. doi: 10.1136/bmjopen-2023-074334 37821130 PMC10582843

[30] Tse T, Fain KM, Zarin DA. How to avoid common problems when using ClinicalTrials.gov in research: 10 issues to consider. BMJ. 2018;361:k1452. 10.1136/bmj.k1452 PMC5968400 29802130

[31] Glanville JM, Duffy S, McCool R, Varley D. Searching ClinicalTrials.gov and the International Clinical Trials Registry Platform to inform systematic reviews: what are the optimal search approaches?. J Med Libr Assoc. 2014;102(3):177–83. doi: 10.3163/1536-5050.102.3.007 25031558 PMC4076126

[32] ClinicalTrials.gov [Internet]. [cited 2025 Aug 27]. Trends and Charts on Registered Studies. Available from: https://clinicaltrials.gov/about-site/trends-charts

[33] Office of the Commissioner. U.S. Food and Drug Administration. FDA; 2025 [cited 2025 Aug 27]. Real-World Evidence. Available from: https://www.fda.gov/science-research/science-and-research-special-topics/real-world-evidence

[34] Jiang Y, Leng Y, Wu Q, Hou Y, Dong X, Zeng C, et al. Understanding the gap between expectations and reality in decentralized clinical trials. NPJ Digit Med. 2025;8(1):408. doi: 10.1038/s41746-025-01811-y 40615632 PMC12227689

[35] Andreoletti M, Senkalfa B, Blasimme A. Ongoing and planned Randomized Controlled Trials of AI in medicine: An analysis of Clinicaltrials.gov registration data [Internet]. medRxiv. 2024. Available from: 10.1101/2024.07.09.24310133

[36] Naslund JA, Deng D. Addressing Mental Health Stigma in Low-Income and Middle-Income Countries: A New Frontier for Digital Mental Health. Ethics Med Public Health. 2021;19:100719. doi: 10.1016/j.jemep.2021.100719 35083375 PMC8786211

[37] Nebie EI, Sawadogo HN, van Eeuwijk P, Signorell A, Reus E, Utzinger J, et al. Opportunities and challenges for decentralised clinical trials in sub-Saharan Africa: a qualitative study. BMJ Open. 2023;13(9):e075903. doi: 10.1136/bmjopen-2023-075903 37739467 PMC10533674

[38] Stewart T. Are Zoom, Skype, Facetime and others alike - HIPAA Compliant? [Internet]. BellMedEx. 2024 [cited 2025 Apr 25]. Available from: https://bellmedex.com/are-video-calling-platforms-hipaa-compliant/

[39] Zum Umgang mit sozialen Medien and Messenger-Diensten. Empfehlungen der FMH [Internet]. Bern: FMH Verbindung der Schweizer Ärztinnen und Ärzte; 2024 May. Available from: https://www.fmh.ch/files/pdf30/empfehlungen_social_media_de.pdf

[40] Sehrawat O, Noseworthy PA, Siontis KC, Haddad TC, Halamka JD, Liu H. Data-driven and technology-enabled trial innovations toward decentralization of clinical trials: opportunities and considerations. Mayo Clin Proc. 2023;98(9):1404–21. 37661149 10.1016/j.mayocp.2023.02.003

