# Supplementary material for: Decentralized clinical trials: A comprehensive analysis of trends, technologies, and global challenges
Source: PLOS Digit Health. 2026 Jan 16;5(1):e0001191. doi: 10.1371/journal.pdig.0001191 (PMC12810901; doi:10.1371/journal.pdig.0001191)
Supplement: S1 Data — (ZIP) [file pdig.0001191.s001.zip › DCTs_dataset_variable codebook.pdf]

Explanation of Computed Columns in the Dataset (see also S2 Table Codebook for further explanations)

“Evaluation”: indicates whether the trial evaluates (=1) or implements (=0) decentralized study elements.

“TrialType”: same as above, indicates whether the trial is an “implementation” or “evaluation” trial.

Three subcode-variables: Subcodes for evaluation studies, indicates what type of (comparative) design the study takes. 1) SubcodeDCTvsCentralized: Does the study compare decentralized and centralized study elements? 2) SubcodeDCTvsDCT: Does the study compare different decentralized elements? 3) SubcodeFeasibilityEfficacy: Does the study evaluate or test the feasibility or efficacy of decentralization? For all three: 1 = yes, 0 = no.

“DigitalHealthTool”: indicates whether a digital health tool was used in the study. 2 = yes, 1 = no,

“NonHealthDigitalTool”: indicates whether a non-health digital tool was used. 1 = yes, 0 = no.

AITools: indicates whether AI/ML-tools were integrated in the study. 1 = yes, 0 = no.

“Enrollment” and “Year”: Actual enrollment numbers and year study started.

“Extracted Countries”: this variable was computed in Python (version 3.13.2). “Extracted Countries” is based on the “Locations” data from ClinicalTrials.gov. A function was applied to the “Locations” data for each row, to extract country names from the more granular records which included strings with information on hospital/university, city, state, and country. For studies with multiple locations in the same country, the country name was only kept once. For studies with multiple locations in different countries, each country name was added to “Extracted Countries”.

“General Category”: this variable was computed in Python (version 3.13.2). “General Category” is based on the “Conditions” data from ClinicalTrials.gov. The research team decided on a list of consolidated “general” condition categories, and a function was designed to iterate over the “Conditions” data for each row, searching for keywords relevant to the general condition categories. If a keyword match was found, the matching general condition category was added to the “General Category” column for the relevant study/row. Trials for which no matching keywords were found were labeled "Other" in the “General Category” column.

“Intervention Category”: this variable was created in Python version (3.13.2). “Intervention Category” is based on the “Interventions” data from ClinicalTrials.gov. A list of general intervention categories was created, largely matching the provided intervention categories ('Behavioral' for 'BEHAVIORAL'; 'Biological' for 'BIOLOGICAL'; 'Device' for 'DEVICE'; 'Drug', for ‘DRUG'; 'Dietary Supplement' for 'DIETARY_SUPPLEMENT’; 'Diagnostic' for

'DIAGNOSTIC_TEST'; 'Procedure' for ‘PROCEDURE’; 'Product' for 'COMBINATION_PRODUCT’; and 'Other' for ‘OTHER’). The “Interventions” data was iterated over for each row, and matching intervention types were added to an intermediary “Found Interventions” column. Only the first intervention category in the “Found Interventions” column was kept for the “Intervention Category” column. If no keyword match was found, the category was set to “Other”.

“Digitalization”: this variable was created in Stata/SE 17.0 to quantify different levels of digitalization in DCTs; it is based on the variables “Digital Health Tools” and “Non-Health Digital Tools”. 1 = No digital tools, 2 = Digital health tool, and 3 = Non-health digital tool.
