# Supplementary material for: Decentralized clinical trials: A comprehensive analysis of trends, technologies, and global challenges
Source: PLOS Digit Health. 2026 Jan 16;5(1):e0001191. doi: 10.1371/journal.pdig.0001191 (PMC12810901; doi:10.1371/journal.pdig.0001191)
Supplement: S1 Table — (DOCX) [file pdig.0001191.s003.docx]

| **Condition Category (High Level)** | **Condition Keywords** |
| --- | --- |
| Pediatric | 'pediatrics', 'pediatric', 'childhood', 'adolescent', 'children', 'neonatal', 'growth', 'parenting', 'infant', 'newborn' |
| Diabetes | 'diabetes', 'glucose', 'glycaemia', 'diabetic', 'diabetes', 'hyperglycemia', 'hypoglycemia' |
| Mental Health | 'delirium', 'grief', 'depression', 'bipolar', 'suicide', 'anxiety', 'emotional', 'depressive', 'mood', 'PTSD', 'stress', 'psychotic', 'hoarding', 'schizophrenia', 'gulf war symptoms', 'gulf war syndrome', 'behavior', 'eating disorder', 'mental', 'psycho', 'burnout', 'opioid', 'cannabis', 'substances', 'addiction', 'alcohol', 'tobacco', 'cigarette', 'smoking', 'nicotine', 'substance' |
| Respiratory | 'ventilator', 'asthma', 'COPD', 'lung', 'respiratory', 'cough', 'pulmonary', 'bronchitis', 'bronchi', 'tuberculosis' |
| Musculoskeletal | 'myasthenia', 'musculoskeletal', 'bone', 'joint', 'arthritis', 'gait', 'claudication', 'knee', 'hip', 'spinal cord', 'palsy', 'limb', 'amputation', 'limb deficiency', 'dystrophy', 'orthopedic', 'prosthesis', 'fracture', 'tear', 'wound', 'ligament', 'fibromyalgia', 'movement', 'tissue', 'meniscectomy', 'rotator cuff', 'shoulder', 'ankle', 'knee', 'hip', 'spinal' |
| Oncological and Hematological | 'cancer', 'tumor', 'carcinoma', 'melanoma', 'lymphoma', 'sarcoma', 'neoplasm', 'glioma', 'myeloma', 'neurofibromatosis', 'leukemia', 'chemotherapy', 'glioblastoma', 'blood', 'anemia', 'hemophilia', 'clonal cytopenia', 'coagulation', 'hematologic', 'hemato', 'sickle cell' |
| Dermatological | 'pressure ulcer', 'dermatitis', 'skin', 'psoriasis', 'acne', 'pemphigoid', 'burn' |
| Pain | 'pain', 'migraine', 'headache' |
| Cardiovascular | 'cardiovascular', 'hypertension', 'heart', 'coronary', 'arrhythmia', 'vascular', 'artery', 'arterial', 'atrial fibrillation', 'myocardial', 'infarction', 'heart failure', 'cardiac', 'arterial disease', 'defibrillator', 'arrythmia', 'arrythmias', 'ventricular', 'bradycardia', 'cardiology', 'cardio', 'pacemaker', 'icd', 'syncope' |
| Organs | 'kidney', 'liver', 'renal', 'cirrhosis', 'transplant' |
| Sleep | 'sleep', 'insomnia', 'apnea', 'narcolepsy' |
| Geriatric | 'geriatric', 'elderly', 'aging', 'old age' |
| Weight and Nutrition | 'malnutrition', 'obesity', 'weight', 'bulimia', 'overweight', 'vitamin', 'mineral', 'nutrient', 'hyperkalemia' |
| Neurological | 'brain', 'tremor', 'Parkinson', 'vision', 'hearing loss', 'deafness', 'blindness', 'cochlear', 'macular degeneration', 'glaucoma', 'cataract', 'macular', 'visual', 'vestibular', 'stroke', 'cerebrovascular', 'dementia', 'Alzheimer', 'neurological', 'neurology', 'epilepsy', 'neuropathy', 'brain injury', 'multiple sclerosis', 'ataxia', 'nervous system', 'cognitive', 'cognition', 'neurologic', 'seizure', 'neurodegenerative', 'neuron', 'neuro', 'nerve', 'nerv', 'autism', ' hyperactivity', 'ADHD', 'intellectual disability', 'Attention-Deficit' |
| Sexual Health | 'sexual', 'erectile', 'libido', 'menopause', 'fertility', 'infertility', 'vaginal', 'gynecologic', 'sexually transmitted', 'gonorrhea', 'syphilis', 'chlamydia', 'herpes', 'HPV', 'hormonal', 'endocrine', 'thyroid', 'adrenal', 'pituitary', 'parathyroid', 'hyperthyroidism', 'hypothyroidism', 'pregnancy', 'maternal', 'postpartum', 'antenatal', 'pregnant', 'abortion', 'fetal', 'breastfeeding', 'urinary', 'bladder' |
| Gastrointestinal | 'gastrointestinal', 'stomach', 'intestine', 'digestive', 'ostomy', 'gut', 'deglutition', 'dyspepsia' |
| Infection and Immunity | 'monkeypox', 'hepatitis c', 'infection','HIV', 'AIDS', 'prophylaxis', 'COVID', 'coronavirus', 'CoV', 'immune', 'cystic fibrosis', 'crohn', 'celiac', 'inflammation', 'autoimmune', 'myositis', 'lupus', 'inflammatory', 'sclerosis', 'sjogren' |
| Clinical Practice | 'surgery', 'ambulatory', 'operation', 'operative', 'anesthesia', 'clinical', 'hospital', 'icu', 'intensive care unit' |
| Other (conditions in dataset that did not match keywords for high level categories) | Healthy Participants, Complication, Military Personnel\|Health Promotion, KRAS P.G12C, Healthy, OSA, Hypotension of Hemodialysis, Accidental Falls, Trauma, Relative Energy Deficiency in Sport, Physical Inactivity, Bullying, Bibliotherapy, Polypharmacy\|Deprescribing\|Telemedicine, Telemedicine, Chronic Diseases, Mass Screening\|Preventive Health Services, Arachnophobia\|Cynophobia\|Ophidiophobia, Vaccine Refusal\|Vaccination, Caregivers\|Parents, Reasoning Training, Critical Illness, No Specific Condition, Telemedicine\|Emergency Medical Service, Telemedicine\|Patient Readmission, Treatment Efficacy, Patient Satisfaction, Patient Discharge\|Drug-Related Side Effects and Adverse Reactions\|Aged\|Health Care Costs, Physical Activity, Multimorbidity\|Physical Deconditioning, Multimorbidity, Comorbidities and Coexisting Conditions, Introduction of Teledermatology Mobile Apps, Telemedicine\|Counseling, Fall, Health Care Utilization, Dental Fear, Chronic Disease, Anticoagulants, Accident, Traffic, Social Distance, Telehealth at the Library\|Control Group, SLE, Telemedicine\|Telehealth, Postural Balance\|Fall, Myopia, Improvement of Treatment Satisfaction, JIA\|Self-Evaluation, CHF, Physical Therapy, Sepsis, Orthodontic Relapse, Symptom Management\|Reduce Treatment Non-adherence, Health-Related Quality Of Life, Post MI, Medication Compliance, AEBT Website With Check-ins\|AEBT Website Without Check-ins, Mucopolysaccharidosis III-A, Hyperekplexia\|CTNNB1 Gene Mutation, GIST, Malignant\|CBT, Telemedicine Evaluation vs Telephone Evaluation, Sepsis\|Trauma, Contraception\|Health Care Utilization, Telemedicine\|Training |
