# Supplementary material for: Decentralized clinical trials: A comprehensive analysis of trends, technologies, and global challenges
Source: PLOS Digit Health. 2026 Jan 16;5(1):e0001191. doi: 10.1371/journal.pdig.0001191 (PMC12810901; doi:10.1371/journal.pdig.0001191)
Supplement: S2 Table — (DOCX) [file pdig.0001191.s005.docx]

**S2 Table. Codebook**

| **Variables and codes** | **Definition** | **Example quote/justification** | **Example keywords and/or tools** |
| --- | --- | --- | --- |
| **VARIABLE 1: AIM OF DECENTRALIZED DESIGN (PRIMARY FOCUS OF DECENTRALIZATION)** | | | |
| 1 = Evaluation | The primary focus of the study is to evaluate if the parts of the study can be delivered in a decentralized way (not necessarily digital). | "This study was designed to determine the validity and reliability of trunk endurance and functional capacity tests applied by the tele-assessment method in healthy individuals" (NCT04899804) | Study specific platforms; digital health application; digital health wearable(s) / remote monitoring tools for vitals/data collection |
| 0 = Implementation | The study implements decentralized elements without evaluation. | "The investigators aim to develop, implement and evaluate a 16-week practice orientated online sports nutrition education and counselling program for female endurance athletes at risk of RED-S" (NCT04959565) | General purpose videoconferencing platforms (e.g., Zoom, Webex, Google Meet, etc.); Whatsapp or other messaging services for communication; email or phone call (smartphone or landline); digital health tools utilized (e.g., Fitbit wearable) but not evaluated |
| **SUBCODES FOR EVALUATION DESIGN** | | | |
| Decentralized vs. centralized study elements | Compares decentralized intervention to centralized intervention | Facebook group arm (access to self-management support program via a Facebook group platform for three months plus the standard care) vs. standard care arm (carry on with the routine diabetes care offered to participants in their health facilities) (NCT04395521) | Experimental approach versus standard treatment; standard care as control group; centralized care or care as usual |
| Decentralized vs. decentralized study elements | Compares different decentralized interventions | "This study evaluates the impact of video communication via telemedicine on the quality of emergency care provided to children by paramedic teams supported by a remote physician in a simulated out-of-hospital setting. Half of the paramedic teams will use a video telemedicine platform for communication with a physician, while the other half will use an audio-only platform." (NCT03742167) | Experimental approach versus standard or comparison treatment (already decentralized); evaluations of experimental approach with varying changes (e.g., audio communication versus audio + visual communication, videoconference versus self-guided therapies) |
| Assessment of feasibility/efficacy only | Evaluates feasibility or efficacy of decentralized approach without comparison | "The study aims to establish if it is possible for people with Cystic Fibrosis to monitor a number of parameters on a daily basis at home which might predict respiratory infections before they have symptoms and which might also predict treatment failures before this is obvious with conventional measures." (NCT02416375) | No comparator; no control group; experimental group only; outcome measure examples: acceptability, feasibility, etc. |
| **VARIABLE 2: DIGITAL HEALTH TOOL** | | | |
| 2 = Digital Health tool | Study uses any digital health technologies specifically designed for health-purposes. | "This study aims to investigate a smartphone-based cardiac rehabilitation program for patients recovering from myocardial infarction. The program focuses on providing patient centric self-monitoring platform enabling patients to take control and be actively involved in their medical care. The program ("Kardia") is a 6-week rehabilitation program enabling patients to track their blood pressure, physical activity and medicine compliance through a mobile application." (NCT03415841) | Wearables and other sensor technology (e.g., health monitoring wearables, smart pill bottles with electronic monitoring, thermometric mats for foot monitoring, multi-sensor home monitoring systems, inertial measurement units for movement tracking), AI/Machine learning-enhanced tools (e.g., AI-based stethoscopes and diagnostic tools, automated speech recognition for monitoring, biosensors with ML-algorithms for monitoring), Health- or study-specific mobile apps (e.g. health management apps, custom-built apps for specific conditions, research apps with specialized functions, disease-specific platforms), integrated digital health platforms (e.g. comprehensive telehealth systems with multiple connected devices, remote monitoring platforms syncing with electronic health records, digital therapeutic platforms combining apps, wearables, and clinical dashboards, virtual reality/specialized interfaces (e.g. VR systems for phantom limb pain or rehabilitation exercises, game-based therapeutic platforms), prosthetics using advanced digital technology and algorithms (EMG-based pattern recognition controllers, etc.). Zoom for Health given it’s specific design for healthcare usage. |
| 1 = No digital health tool | Study does not implement any digital health tools as defined above. | "the trial examines whether a brief, behavioral activation (BA) treatment delivered via telemedicine is as effective as the same treatment delivered in person" (NCT04153864) | Video conferencing (e.g. Zoom, Microsoft Teams, Skype), electronic consent (e.g. electronic consent platforms, digital signature tools, web-based remote consent), data collection platforms (e.g. mobile apps for questionnaires, electronic patient-reported outcomes (ePRO), online screening platforms), communication tools (e.g. basic apps for communicating with study providers such as e.g. Whatsapp, email systems, messaging platforms), online portals for study information (e.g., web-based data entry systems) Additional non-digital tools without remote monitoring abilities (conventional health devices): BP cuff, pulse oximeter, non-digital scale, cochlear implant, ICD, spirometer, inhaler, etc. (conventional health tools but non-digital) |
| **VARIABLE 3: DIGITAL STUDY DELIVERY COMPONENTS (for trials *not* including digital health tools)** | | | |
| 1 = Digital delivery of study elements | Study use digital platforms to deliver elements of the study. | "The SLEEP-HD study is a randomized open-label clinical trial to compare two types of treatment for insomnia in participants who have end-stage renal disease on HD, and who have been diagnosed with chronic insomnia. The two types of treatment involved in the study are Cognitive Behavioral Therapy for Insomnia (CBT-I) or treatment with a drug (trazodone vs placebo) [...] The CBT-I sessions will be delivered by a therapist face-to-face with the patient via a fully interactive video telehealth platform." (NCT03534284) | Video conferencing (e.g. Zoom, Microsoft Teams, Skype), electronic consent (e.g. electronic consent platforms, digital signature tools, web-based remote consent), data collection platforms (e.g. mobile apps for questionnaires, electronic patient-reported outcomes (ePRO), online screening platforms), communication tools (e.g. basic apps for communicating with study providers such as Whatsapp, email systems, messaging platforms), online portals for study information (e.g., web-based data entry systems) |
| 0 = No digital elements included | Study relies on non-digital methods/no digital tools mentioned. | "The purpose of the study is to test if Acceptance and Commitment Therapy (ACT), a behavioral intervention designed to increase psychological flexibility in the face of challenges, reduces anxiety associated psychological distress in dementia caregivers compared to the control group who will receive self-help and educational materials [...] All caregivers randomized to the mEUC group will receive a mailed packet containing 1) a letter from the Co-PIs thanking them for participating, 2) printed selections from of the NIH Alzheimer's caregiving website" (NCT04780178) | Physical delivery (e.g., home delivery of medications, at-home drop-off of study materials, smart medication lockers), sample collection (e.g., mailed blood spot collection kits, physical specimen collection kits, home-collected samples), communication (e.g., landline telephone calls for check-ins, postal mail, paper forms), food/nutrition (e.g., home-delivered meals, produce delivery, gift cards to grocery stores), clinical services (e.g., mobile clinic visits to participant homes, home-based care team assessments), educational materials (e.g., exercise manuals, printed instructions, paper diaries for tracking ), medical devices (e.g., physical stimulator devices, conventional medical equipment delivered to home) |
