# Supplementary material for: Decentralized clinical trials: A comprehensive analysis of trends, technologies, and global challenges
Source: PLOS Digit Health. 2026 Jan 16;5(1):e0001191. doi: 10.1371/journal.pdig.0001191 (PMC12810901; doi:10.1371/journal.pdig.0001191)
Supplement: S3 Table — (DOCX) [file pdig.0001191.s006.docx]

**S3 Table. List of trial location countries with country status**

| **Country** | **Trial Count** (includes multi-country trials; n =/ 1370) | **Country Status** ([WB Income Level](https://datahelpdesk.worldbank.org/knowledgebase/articles/906519)) |
| --- | --- | --- |
| United States of America | 799 | High |
| Canada | 77 | High |
| United Kingdom | 69 | High |
| France | 66 | High |
| Spain | 44 | High |
| Germany | 38 | High |
| Italy | 38 | High |
| Brazil | 26 | Upper Middle |
| Netherlands | 22 | High |
| Sweden | 20 | High |
| Denmark | 18 | High |
| Norway | 17 | High |
| Turkey | 16 | Upper Middle |
| Australia | 15 | High |
| Singapore | 14 | High |
| Belgium | 14 | High |
| Austria | 13 | High |
| Hong Kong | 13 | High |
| China | 13 | Upper Middle |
| Israel | 10 | High |
| Korea | 10 | High |
| Ireland | 10 | High |
| Finland | 7 | High |
| Switzerland | 7 | High |
| Greece | 7 | High |
| Portugal | 7 | High |
| South Africa | 6 | Upper Middle |
| India | 6 | Lower Middle |
| Taiwan | 5 | High |
| Iceland | 5 | High |
| Russian Federation | 4 | High |
| Romania | 3 | High |
| Czechia | 5 | High |
| Chile | 3 | High |
| Slovenia | 3 | High |
| Hungary | 3 | High |
| Malaysia | 3 | Upper Middle |
| Poland | 3 | High |
| Luxembourg | 2 | High |
| Lithuania | 2 | High |
| Colombia | 2 | Upper Middle |
| Thailand | 2 | Upper Middle |
| Vietnam | 2 | Lower Middle |
| Peru | 2 | Upper Middle |
| Mexico | 2 | Upper Middle |
| Uganda | 2 | Low |
| Tanzania | 2 | Lower Middle |
| Tunisia | 2 | Lower Middle |
| Nepal | 1 | Lower Middle |
| Gabon | 1 | Upper Middle |
| Kyrgyzstan | 1 | Lower Middle |
| Rwanda | 1 | Low |
| Paraguay | 1 | Upper Middle |
| Slovakia | 1 | High |
| Latvia | 1 | High |
| United Arab Emirates | 1 | High |
| Pakistan | 1 | Lower Middle |
| New Zealand | 1 | High |
| Cyprus | 1 | High |
| Lebanon | 1 | Lower Middle |
| Cambodia | 1 | Lower Middle |
| Iran | 1 | Upper Middle |
| Ghana | 1 | Lower Middle |
| Nigeria | 1 | Lower Middle |
| Niger | 1 | Low |
| Sierra Leone | 1 | Low |
| Mozambique | 1 | Low |
| Côte D'Ivoire | 1 | Lower Middle |
| Cameroon | 1 | Lower Middle |
| Puerto Rico | 1 | High |
| Zambia | 1 | Lower Middle |
| Argentina | 1 | Upper Middle |
| Eswatini | 1 | Lower Middle |
| Lesotho | 1 | Lower Middle |
| Kenya | 1 | Lower Middle |
