# Supplementary figures and images for: Decentralized clinical trials: A comprehensive analysis of trends, technologies, and global challenges
Source: PLOS Digit Health. 2026 Jan 16;5(1):e0001191. doi: 10.1371/journal.pdig.0001191 (PMC12810901; doi:10.1371/journal.pdig.0001191)

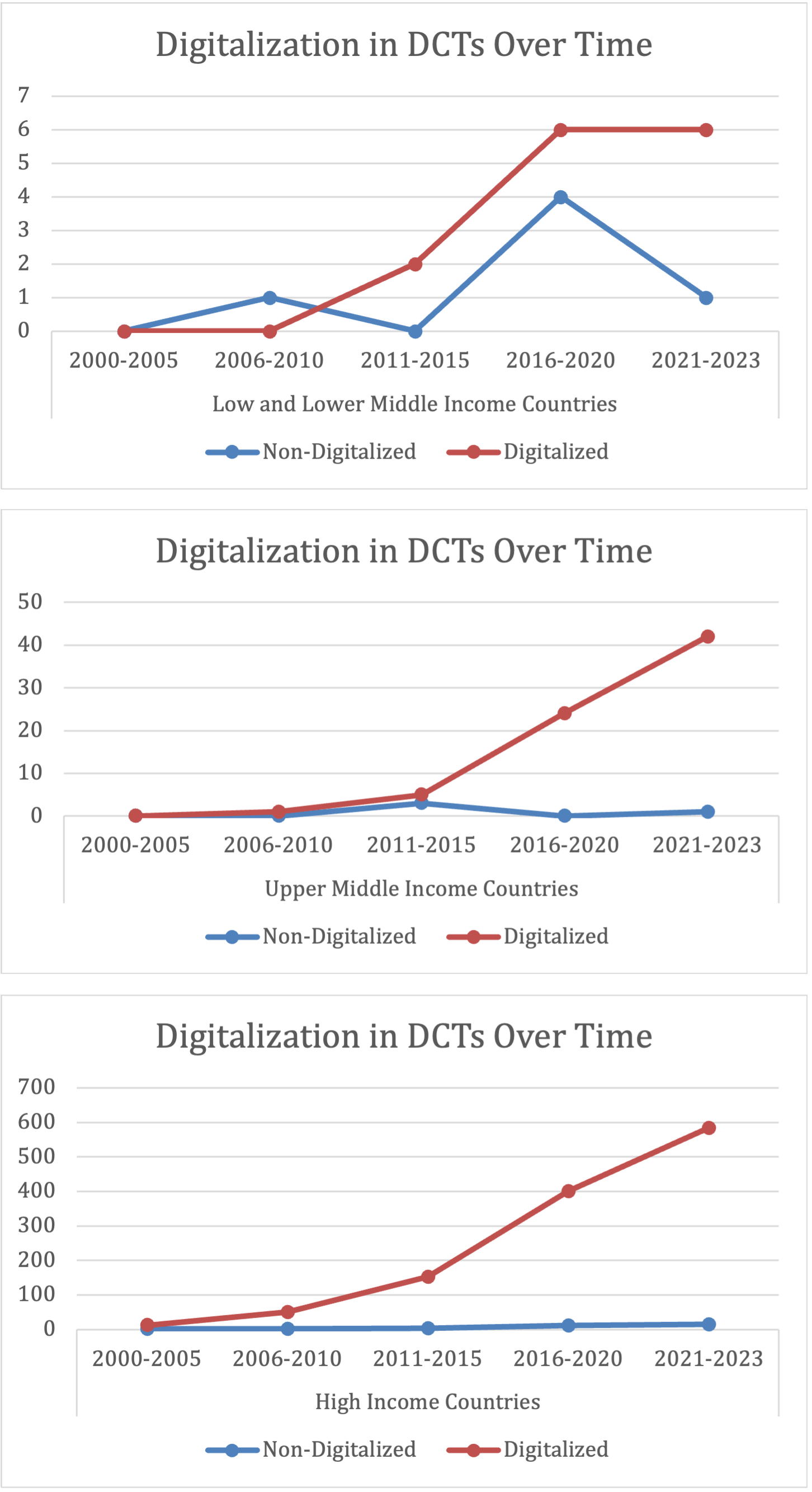

Supplement: S3 Fig — (TIF) [file pdig.0001191.s007.tif]

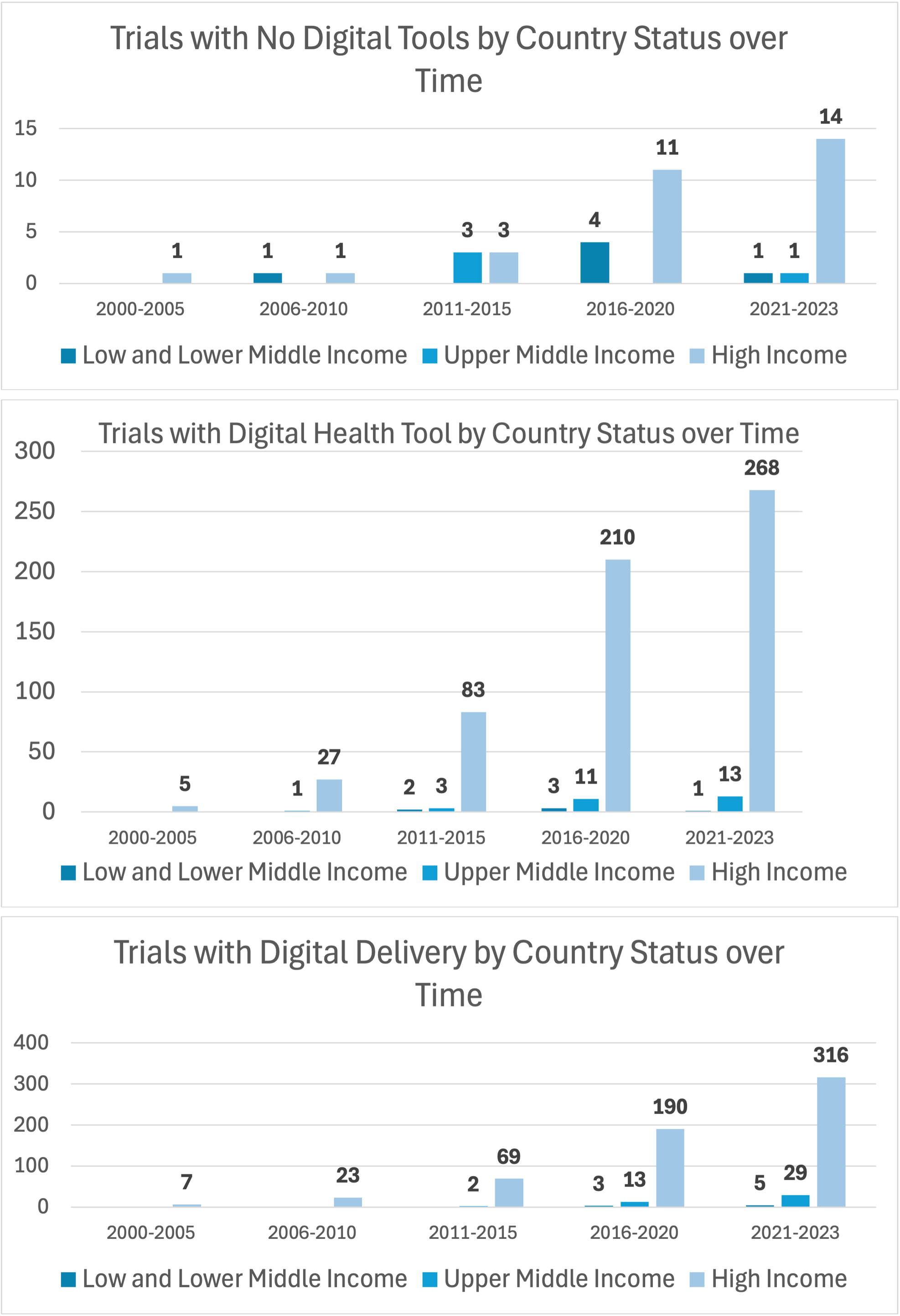

Supplement: S4 Fig — (TIF) [file pdig.0001191.s008.tif]
