# Supplementary material for: Decentralized clinical trials: A comprehensive analysis of trends, technologies, and global challenges
Source: PLOS Digit Health. 2026 Jan 16;5(1):e0001191. doi: 10.1371/journal.pdig.0001191 (PMC12810901; doi:10.1371/journal.pdig.0001191)
Supplement: S4 Table — (DOCX) [file pdig.0001191.s009.docx]

**S4 Table. Exemplars illustrating variation of DCTs across decentralization and digitization dimensions**

|  |  |  | Decentralization Dimension | |  |
| --- | --- | --- | --- | --- | --- |
|  |  | Implementation Trials | | Evaluation Trials | |
| Digitization Dimension | Non-digital | **Percent of Trials:** 1.9%  **Exemplar:**  ClinicalTrials.gov ID: NCT02910427  Trial Description: investigate impacts of different mineral-enriched salts on neurological health of stroke survivors; salts provided to participants to consume at home, as part of their daily lives | | **Percent of Trials:** 3.1%  **Exemplar:**  ClinicalTrials.gov ID: NCT03575026  Trial Description: evaluate effectiveness of home-based caregiver delivery of a music-with-movement intervention for dementia patients | |
|  | Digital Delivery | **Percent of Trials:** 14.4%  **Exemplar:**  ClinicalTrials.gov ID:  NCT02494050  Trial Description: compares efficacy of three behavioral interventions for treating anhedonia, with treatment sessions completed over the phone | | **Percent of Trials:** 27.9%  **Exemplar:**  ClinicalTrials.gov ID: NCT00668551  Trial Description: investigate feasibility and efficacy of telemedicine appointments for lowering barriers to care for rural Parkinson’s disease patients | |
|  | Digital Health Tools | **Percent of Trials:** 9.7%  **Exemplar:**  ClinicalTrials.gov ID: NCT04453072  Trial Description: evaluate effectiveness of an addiction-based behavioral weight loss intervention, delivered via a specialized app | | **Percent of Trials:** 43.0%  **Exemplar:**  ClinicalTrials.gov ID: NCT05563597  Trial Description: evaluate feasibility and acceptability for participants of tracking cognitive performance via a validated online cognition assessment tool | |
